# Ultrasound-assisted recovery of blackcurrant press cake anthocyanins: Antioxidant and anti-inflammatory properties, bioaccessibility, and application in functional gummies

**DOI:** 10.1016/j.fochx.2025.102285

**Published:** 2025-02-18

**Authors:** Emma Brennan, Carolina Girotto Pressete, Nima Mohammadi, Lusânia Maria Greggi Antunes, Qixiang Shang, Jihang Chen, Jason Bennett, Marcelo Franchin, Daniel Granato

**Affiliations:** a Bioactivity & Applications Laboratory, Department of Biological Sciences, Faculty of Science and Engineering, University of Limerick, V94 T9PX Limerick, Ireland; b Department of Clinical Analysis, Toxicology and Food Science, School of Pharmaceutical Sciences of Ribeirão Preto, University of São Paulo, Ribeirão Preto, Brazil; c School of Medicine, The Chinese University of Hong Kong, Shenzhen, People's Republic of China

**Keywords:** Circular economy, Innovative extraction technologies, Flavonoids, Food models, Chemical stability, Human erythrocytes, Molecular docking

## Abstract

Blackcurrant press cake (BPC) anthocyanins were recovered using ultrasound-assisted extraction, and the optimal BPC extract was tested for its antioxidant capacity using chemical and biological assays and applied in a functional food model. Extraction at 400 W for 10 min followed by freeze-drying rendered an extract rich in polyphenols (47.83 mg GAE/g), where delphinidin-3-rutinoside, delphinidin-3-glucoside, cyanidin-3-rutinoside, and cyanidin-3-glucoside accounted for 75 % of total phenolics. When the reverse process of protonation/deprotonation was performed (pH 10 to pH 2), anthocyanins exhibited 79.3 % reversibility. The BPC extract inhibited human plasma oxidation (5052 mg AAE/g) and decreased intracellular reactive oxygen species generation by 54 % in erythrocytes and 45 % in LPS-stimulated THP-1 macrophage-like cells. BPC extract exhibited antiproliferative activity and cytostatic effect on HepG2 cells in monoculture at 100–250 μg/mL. Gummies added with BPC extract had a sensory acceptability of 75 %, but bioactive compounds had a low bioaccessibility.

## Highlights

• Optimal ultrasound-assisted extraction was at 400 W/10 min using water/ethanol (60 %).
• BPC extract decreased intracellular ROS by 54 % in erythrocytes and 45 % in THP-1 cells.
• BPC extract exhibited antiproliferative activity and cytostatic effect on HepG2 cells.
• Gummies added with BPC extract had a sensory acceptability of 75 %.
• The bioaccessibility rate of antioxidants was reduced after in vitro digestion.

## Graphical abstract

**Figure:**
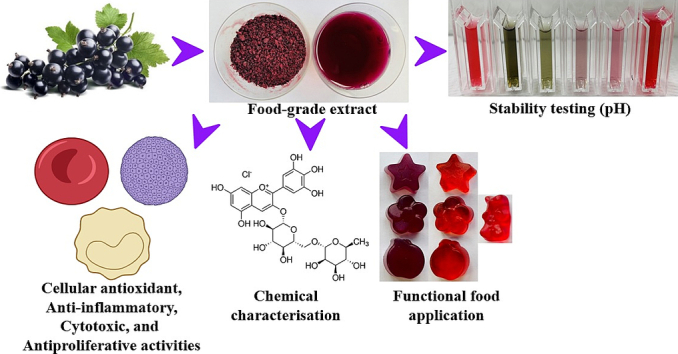

## 1 Introduction

The food industry's increasing emphasis on sustainable practices and consumer demand for natural ingredients has led to exploring novel methods to minimize waste and enhance product functionality. Blackcurrant press cake (BPC), a by-product of juice production, is an underutilized resource with a high content of bioactive compounds, including anthocyanins, dietary fiber, and essential minerals (Schieber, 2017). Despite its potential, BPC is often discarded or used for low-value applications, representing a missed opportunity for creating high-value functional food products that align with the principles of the circular economy. The circular economy model advocates for extending resource lifecycles, minimizing waste, and promoting environmental sustainability (Kandpal et al., 2024; Patwa et al., 2021).

Anthocyanins, particularly delphinidin-3-rutinoside, delphinidin-3-glucoside, and cyanidin-3-rutinoside, are abundant in blackcurrants (*Ribes nigrum* L.) and are well-known for their potent antioxidant and anti-inflammatory properties (Šimerdová et al., 2021). These compounds play a significant role in neutralizing reactive oxygen species (ROS), thereby mitigating oxidative stress, a key factor in the progression of chronic diseases such as cardiovascular disease and cancer (Ali Redha et al., 2024; Rahaman et al., 2023). Natural antioxidants, like those found in BPC, offer a promising alternative to synthetic additives, responding to the growing consumer preference for clean-label products while enhancing the nutritional value of foods.

Incorporating BPC into food products not only supports health outcomes by providing functional ingredients but also addresses key sustainability challenges. This approach aligns with the circular economy by repurposing what is typically considered waste into valuable nutritional resources (Salvatore et al., 2024). Integrating BPC into functional food products marks a transformative step toward more sustainable food systems, demonstrating how scientific advancements in food processing can drive the global shift toward a circular economy, where waste is reimagined as a resource rather than a liability. Furthermore, using BPC supports food manufacturers in meeting the rising demand for sustainable, health-promoting products that offer both environmental and health benefits (Tulloch et al., 2022).

However, translating the antioxidant potential of BPC from in vitro chemical assays to real-world applications poses challenges. While in vitro antioxidant assays, such as DPPH and FRAP assays, are commonly used to measure antioxidant activity, they are not always reflective of in vivo conditions (Granato, 2023). The complexity of the food matrix and the digestion process can alter the bioavailability and efficacy of these bioactive compounds, which necessitates further investigation using more biologically relevant models, such as cellular antioxidant activity (CAA) assays (Munteanu & Apetrei, 2021). The CAA method provides a more accurate representation of how antioxidants interact with ROS in living cells, offering valuable insights into their potential health benefits in real-world consumption scenarios (Granato, 2023).

This study offers a novel perspective on BPC valorisation by integrating ultrasound-assisted anthocyanin extraction, response surface methodology (RSM), and advanced bioactivity evaluations, including molecular docking, cytotoxicity, and antiproliferative assays. Unlike previous research focused on toxicological properties and chemical composition (dos Santos Lima et al., 2024a; Pap et al., 2021a; Šimerdová et al., 2021), it translates these findings into a functional food application—a gummy enriched with BPC anthocyanins—providing insights into both chemical and biological properties. By bridging the gap between in vitro bioactivity and real-world functionality, this study highlights BPC's untapped potential in sustainable food systems.

## 2 Materials and methods

### 2.1 Chemical reagents

Phosphoric acid (98 %), trichloroacetic acid (TCA), methanol (HPLC grade), lipopolysaccharide (LPS), dimethyl sulfoxide (DMSO), RPMI-1640 medium, Folin-Ciocalteu's phenol reagent, magnesium chloride, α-amylase from porcine pancreas (14 U/mg, A3176- 500KU), pepsin from porcine gastric mucosa (≥ 500 U/mg 77,160-100G), bile salts (B8756-100G), pancreatin from porcine pancreas (P1750-100G), ferric chloride hexahydrate, 3-(4,5-dimethylthiazol-2-yl)-2,5-diphenyl tetrazolium bromide (MTT), 2′,7′-dichlorofluorescein diacetate (DCFH-DA), and 2,2′-azobis(2-methylpropionamidine) dihydrochloride (AAPH) were acquired from Merck KGaA (Darmstadt, Germany). Sodium chloride, calcium chloride, sodium hydroxide, ammonium acetate, and hydrogen peroxide at 30 % were purchased from Scientific Laboratory Supplies (Dublin, Ireland). HPLC standards (>98 % purity) of delphinidin-3-glucoside, delphinidin-3-rutoside, cyanidin-3-glucoside, and cyanidin-3-rutoside were purchased from Extrasynthese (Genay, France). The following ingredients were used for producing the gummies: beef gelatine powder (Dr. Oetker, Dublin, Ireland), xylitol and inulin extracted from chicory root (fructooligosaccharides content of 89 %, Bulk Sports Supplements Limited, Essex, United Kingdom), and blueberry flavouring drops (My Protein, Manchester, United Kingdom).

### 2.2 Blackcurrant press cake and extraction

Dried blackcurrant press cake (moisture content below 10 %) was obtained from a berry processing company in Suomussalmi, East Finland. The centesimal composition of the BPC has been reported in our previous work (dos Santos Lima et al., 2024a): 67 % dietary fiber, 3.5 % ash, 2.5 % lipids, 12 % protein, and 11 % other carbohydrates.

The extraction of anthocyanins used a green solvent (ethanol and water at a 60:40 *v*/v ratio) and a solid-to-solvent ratio of 1.5:30 (g/mL). Time (5, 7.5, and 10 min) and ultrasound power (250, 325, and 400 W) were the independent factors studied at two levels (−1 and + 1) and one central point (0, 0), totalling 5 extraction conditions. The ultrasonic liquid processor (model CL-334, Thermo Fisher Scientific, Karlsruhe, Germany) operated using a frequency of 20 kHz. Extractions (100 mL, 3 independent replicates) were performed using an ice bath and aluminium foil to cover the flask and avoid photo- and thermal degradation of anthocyanins during sonication. The extracts were vacuum filtered, and the extracts were analysed immediately.

### 2.3 Composition, colour attributes, and antioxidant capacity

The total phenolic content (TPC) was assessed through UV-VIS spectrophotometry using the Folin-Ciocalteu method for each extract (Margraf et al., 2015). An analytical curve was prepared using gallic acid (linearity: 15–160 mg/L, R^2^ = 0.998) and the results were expressed in mg of gallic acid equivalent per gram (mg GAE/g). The total monomeric anthocyanin content (TAC) was assessed by the pH differential method based on the structural transformations that occur at pH 1.0 and 4.5 (Lee et al., 2005). The molar absorption (26,900 L x cm^−1^ x mol^−1^) and molecular weight (449.2 g/mol) of cyanidin-3-glucoside were adopted. The results were expressed as mg of cyanidin-3 glucoside equivalent per gram (mg C3G/g).

The instrumental colour of each extract was analysed using two methodologies: maximum absorbance at 420 nm (yellow pigments), 520 nm (red pigments), and 620 nm (blue pigments) were recorded using a Synergy H1 microplate reader (BioTek, Winooski, USA), and the colour intensity (CI) was estimated using Eq. 1:

(1) $$\mathrm{CI}={\mathrm{Abs}}_{42\text{0nm}}+{\mathrm{Abs}}_{52\text{0nm}}+{\mathrm{Abs}}_{62\text{0nm}}$$

The CIE L* (lightness [0 = black, 100 = white]), a* (−a* = greenness, +a* = redness), and b* (−b* = blueness, +b* = yellowness) values were recorded using a CR-400 Konica Minolta colourimeter (Konica Minolta Business Solutions, Ramsey, NJ, USA). A D65 optical sensor, 0° geometry, and 10° angle of vision were used in the analyses. These L*, a*, and b* values were inputted to a colour converter (nixsensor.com) to obtain RGB colour transformations.

A synthetic food-grade colouring solution (1 mL/100 mL water) composed of Ponceau 4R E124, and Sunset Yellow E110 pigments (Goodall's of Ireland, Valeo Foods, Dublin, Ireland) was used as a standard. Additionally, a natural elderberry pigment solution (Creative Flavours Ireland) was also analysed for comparison purposes.

Extracts' antioxidant capacity was estimated using the DPPH and FRAP assays following the experimental conditions adopted elsewhere (Santos et al., 2020). For the DPPH assay, a methanolic solution at 0.10 mmol/L was used, and the absorbance was recorded at 517 nm. Ascorbic acid was used as the calibrator for the analytical curve (3–60 mg/L, R^2^ = 0.997), and the results were expressed as mg of ascorbic acid equivalent per gram (mg AAE/g). For FRAP, the working solution was prepared using a 300 mmol/L sodium acetate solution at pH 3.6, TPTZ at 10 mmol/L and FeCl_3_.6 H_2_O at 20 mmol/L. Absorbance was recorded at 593 nm. Ascorbic acid was used as the calibrator for the analytical curve (11–88 mg/L, R^2^ = 0.999), and the results were expressed as mg AAE/g. Extracts' ability to mitigate ROS in human plasma was evaluated using a standardised procedure (Mohammadi, Farrell, et al., 2024). Fresh blood was collected from a healthy (BMI < 25 kg/m^2^, total cholesterol level of 133 mg/dL and total triglyceride level of 56 mg/dL) after clearance from the Research Ethics Committee (protocol 2023_02_01_S&E) was obtained. Plasma was obtained after centrifugation (900 *xg*, 10 min), and lipid peroxidation was induced using a 150 mmol/L CuCl_2_ solution. The absorbance was monitored at 245 nm for 120 min, and ascorbic acid was used to generate an analytical curve (0 to 100 mg/L, R^2^ = 0.999). The results were expressed as mg AAE/g.

### 2.4 Identification of the optimal extraction conditions

The results were utilized to generate multiple regression models via Response Surface Modelling (RSM) using Eq. 2. This allowed for the assessment of the effects of extraction time (x_1_) and ultrasound power (x_2_) on the colour attributes, TPC, TAC, and antioxidant capacity of the extracts. The fitting quality was analysed using the determination coefficient (R^2^) and adjusted R^2^ when the regression equation was obtained with significant (*p* < 0.10) coefficients.

(2) $$E\left(y\right)=\beta_{0}+\beta_{1}x_{1}+\beta_{2}x_{2}+\beta_{3}x_{1}x_{2}$$

In addition to employing RSM, inferential statistical analyses, including one-way analysis of variance (ANOVA) and Tukey's post-hoc test, were conducted to identify significant differences (*p* < 0.05) between extraction conditions and to determine the conditions yielding the highest TPC, TAC, and antioxidant capacity – the so-called “optimal extract”. All statistical calculations were performed using TIBCO Statistica v.13 (TIBCO Statistica Inc., Palo Alto, CA, USA).

The conditions that yielded the highest total anthocyanin content and antioxidant capacity were used to obtain the optimal BPC extract. Briefly, 600 mL of extract was produced in two batches using the same conditions (ultrasonication employing ethanol and water at a 60:40 *v*/v ratio and a solid-to-solvent ratio of 1.5:30 g/mL). After filtration, the solutions were centrifuged for 5 min at 1000 x*g* to eliminate solid impurities. Considering the bioassays and gummy development, the ethanol was then evaporated using a rotary evaporator under vacuum pressure at 40 °C to yield the concentrated extract, and distilled water was used to compensate for the ethanol removal. This original extract was used for physicochemical analysis (2.3, 2.5.1) and incorporation into gummy formulations (section 2.7.1). The optimal extract was freeze-dried for 24 h at −57 °C and 0.0010 mbar for bioactivity testing and cell-based experiments.

### 2.5 Characterisation of the blackcurrant press cake optimal extract

#### 2.5.1 Anthocyanin composition, colour, and chemical stability toward pH

The optimal BPC extract's colour attributes were analysed according to section 2.3. The chemical stability and reversibility of anthocyanins were assessed using the method described by Mohammadi, Franchin, et al. (2024) between pH 2 and pH 10. The optimal BPC extract (100 mL, absorbance of 0.80 at 525 nm) was added with HCl (1 mol/L) until pH 2 was reached. Then, the reverse titration using NaOH (2.0 mol/L) was performed until pH 4, 6, 8, and 10 were obtained. Aliquots (500 μL) were collected at different pH values, so the colour attributes (section 2.3) and the antioxidant capacity measured by the FRAP and DPPH assays (section 2.3) were performed. The UV–Vis spectrum (λ = 450–700 nm) of the optimal BPC extract at different pH values was also recorded. The reversibility rate of anthocyanins, expressed in percentage, was calculated by the ratio between the absorbance (525 nm) of the BPC extract at final pH 2 (e.g., after adding NaOH) and the solution at pH 2 (initial extract). The effects of pH on the reversibility of antioxidant compounds in the optimal BPC extract was also calculated by the ratio between the final antioxidant capacity value measured by FRAP and DPPH assays at final pH 2 (e.g., after adding NaOH) and the solution at pH 2 (initial extract).

Anthocyanins, including cyanidin-3-rutinoside, cyanidin-3-glucoside, delphinidin-3-glucoside, and delphinidin-3-rutinoside, were quantified using high-performance liquid chromatography (HPLC; Agilent 1200 Infinity Series, Agilent Technologies, Santa Clara, CA, USA). A Telos LU C18 column (5 μm, 150 × 4.6 mm) was employed, with an injection volume of 30 μL. The mobile phase consisted of ultrapure water acidified with 1 % phosphoric acid (solvent A) and acetonitrile (solvent B), following these gradient conditions: 92 % A at 0 min, 88 % A at 3 min, 86 % A at 25 min, 84 % A at 28 min, and 92 % A at 30 min. Anthocyanins were detected using a diode array detector (DAD) set at 520 nm, and quantification was achieved through analytical curves (linearity: 5–40 mg/L, R^2^ = 0.999) prepared with pure standards. The optimal extract was analysed in triplicate.

### 2.6 Cellular antioxidant, anti-inflammatory, cytotoxic and antiproliferative activity in human-derived cell lines

#### 2.6.1 Erythrocyte cellular antioxidant activity

Human red blood cells (RBC) were obtained after plasma collection (section 2.3). Aliquots of 100 μL RBC suspension (hematocrit 20 % in PBS at pH 7.4) were mixed with 100 μL of PBS (positive control – maximum oxidation), quercetin (negative control), or the liquid dealcoholised optimal BPC extract diluted 20- and 40-fold with PBS in 2 mL polyethylene microtubes. Mixtures were incubated at 37 °C with shaking at 150 rpm (Orbital Shaker-Incubator ES-20, Biosan, Riga, Latvia). To induce oxidation, 200 μL of AAPH (200 mmol/L in PBS) was added, followed by incubation at 37 °C for 2 h with shaking at 150 rpm. After AAPH treatment, mixtures were centrifuged at 1200 ×*g* for 10 min. The supernatant was discarded, and the precipitate was washed with 400 μL PBS and centrifuged at 1200 ×*g* for 3 min. Cells were resuspended in 400 μL DCFH-DA solution (10 μmol/L), and 300 μL aliquots were incubated at 37 °C in the dark for 20 min. Intracellular ROS levels were measured by fluorescence (excitation 485 nm, emission 520 nm) using a microplate fluorometer. The negative control contained RBCs with PBS (no AAPH), and the positive control contained RBCs treated with AAPH. Eq. 3 was used to calculate intracellular ROS generation in each treatment. This formula normalizes the ROS levels in the samples by comparing them to the baseline (negative control) and the maximum ROS levels (positive control). The protocol received approval from the University of Limerick Research Ethics Committee - Faculty of Science and Engineering (Approval No. 2023_02_01_S&E). Informed written consent was obtained from the blood donor.

(3) $$\mathrm{ROS}\;\text{generation}\;\left(\%\right)=\left[\left(F_{\text{Sample}}–F_{\text{Blank}}\right)/\left(F_{\text{Positive Control}}–F_{\text{Blank}}\right)\right]\;x\;100$$

#### 2.6.2 THP-1 cell culture and cell viability

Human THP-1 monocytic cells (TIB-202™, ATCC, Virginia, USA) were maintained in RPMI-1640 medium supplemented with 10 % fetal bovine serum (FBS), 2 mmol/L l-glutamine, antibiotics (150 U/mL penicillin, 200 U/mL streptomycin), and 0.05 mmol/L 2-mercaptoethanol in an incubator at 37 °C in the presence of 5 % CO_2_. Differentiation of THP-1 monocytes into THP-1 macrophage-like cells was performed as previously described (Giambelluca et al., 2022). Briefly, THP-1 monocytic cells were seeded at a density of 20,000 cells/well in 96-well plates containing RPMI-1640 medium supplemented with 100 nmol/L phorbol 12-myristate 13-acetate (PMA) for 72 h. Following PMA differentiation, macrophage-like cells were cultured in RPMI-1640 medium for 24 h before any treatment.

THP-1 macrophages were treated with the optimal extract at 1, 10, 100, 1000, and 2000 μg/mL concentrations. For the control group, RPMI was added. After 24 h, the supernatant was removed, and the cells were washed with 200 μL of 1× PBS and 200 μL of RPMI containing MTT at 0.3 mg/mL was added. After 3 h, the supernatant was removed, and 200 μL of DMSO was added. Absorbance was measured at 540 nm using a microplate reader (Microplate Spectrophotometer, BioTekEon®, Winooski, USA) (Mohammadi, Franchin, et al., 2024; Mosmann, 1983).

##### 2.6.2.1 Effects of blackcurrant optimal extract on ROS production and Nfr2 activation

THP-1 macrophages were pre-treated with the optimal extract at 100, 300, and 1000 μg/mL concentrations diluted in DCFH-DA (5 mmol/L), and after 30 min, 100 ng/mL LPS was added, and the cells were incubated at 5 % CO_2_ and 37 °C for 6 h. Afterwards, the supernatant was removed, and 100 μL of Hanks' working solution was added. Fluorescence reading was measured (emission at 538 nm and excitation at 485 nm) using a microplate reader (Synergy H1, BioTek, Winooski, VT, USA) (Escher et al., 2018; Mohammadi, Franchin, et al., 2024; Murphy et al., 2022). The Human Nrf2 ELISA Kit from RayBio® (#ELH-NRF2, Peachtree Corners, USA) was used to quantify nuclear factor erythroid 2-related factor 2 (Nfr2) in the supernatant collected from cells. Absorbance was measured at 450 nm using a microplate reader (Microplate Spectrophotometer, BioTekEon®, Winooski, USA) (Mohammadi, Franchin, et al., 2024).

##### 2.6.2.2 Effects of blackcurrant optimal extract on pro-inflammatory cytokine secretion

THP-1 macrophages were pre-treated with Blackcurrant extract at 100 and 300 μg/mL concentrations, and after 30 min, 100 ng/mL LPS was added. The cells were then incubated at 5 % CO_2_ and 37 °C for 6 h for TNF-α or 24 h for IL-6. Afterwards, the supernatant was collected, and the cytokines were quantified by ELISA using DuoSet ELISA Development Systems kits (TNF-α, R&D Systems #P377304, Minneapolis, MN, USA; IL-6, R&D Systems #P388437, Minneapolis, MN, USA), according to the manufacturer's instructions.

#### 2.6.3 Anti-proliferative activity and cytotoxicity in other human-derived cell lines

The hepatocellular carcinoma HepG2 cell line and colorectal adenocarcinoma HT-29 were purchased from the Rio de Janeiro Cell Bank. The human umbilical vein endothelial HUVEC cell line was purchased from ATCC (American Type Culture Collection). The cells were grown in Dulbecco's Modified Eagle's Minimum Essential Medium (DMEM) (Sigma, CA, USA) containing 10 % fetal bovine serum and 1 % antibiotic-antimycotic (10,000 units/mL penicillin, 10,000 μg/mL streptomycin and 25 μg/mL amphotericin B). The cells were kept in a 3110 Series II CO_2_ water-jacketed incubator (Thermo Fisher Scientific; Carlsbad, CA, USA) at 37 °C with a 5 % CO_2_ atmosphere and 96 % relative humidity. All culture procedures followed the recommendations proposed by Bal-Price and Coecke (2011). The blackcurrant optimal extract was solubilised in DMSO-20 %, and MiliQ water (80 %), and the stock solution (10,000 μg/mL) was stored at −20 °C until use. The extract was solubilised in a fresh culture medium at final concentrations immediately before treatment.

##### 2.6.3.1 Tumour spheroids culture

HepG2 and HT-29 tumour spheroids were obtained using the agarose coated-overlay method, according to Friedrich et al. (2009a). Briefly, 96-well plates (Greiner Bio-One; Monroe, NC, USA) were previously coated with 2 % normal melting point agarose (NMP; Invitrogen —Thermo Fisher Scientific; Carlsbad, CA, USA) in PBS. After solidification, HepG2 cells (1.5 × 10^3^) and HT-29 (4.0 × 10^3^) suspended in a complete culture medium were added to each well using a micropipette. The plates were transferred to the incubator and held immobile for 96 h for tumour spheroids formation (called initiation). The integrity of the spheroid and growth kinetics were monitored initially for 72 h of exposure with optimal BPC extract and, subsequently, every 48 h through analysis of images acquired under a phase contrast microscope (Carl Zeiss, Germany).

##### 2.6.3.2 Cell viability assay (monoculture)

The resazurin assay was performed according to Page et al. (1993). Cells were seeded in 96-well plates at 1 × 10^4^ cells (HepG2, HT-29 and HUVEC) per well for 24 h treatments. For 72 h treatments, HepG2 and HT-29 cells were seeded in 96-well plates at 1 × 10^4^ per well and HUVEC cell at 5 × 10^3^ per well. The cells were treated with DMEM (NC-negative control), 0.08 % *v*/v DMSO (SC-solvent control), or optimal BPC extract (5–250 μg/mL) for 24 and 72 h to determine IC_50_ values. Next, a resazurin working solution (0.15 mg/mL in PBS) was added to 15 % of the final volume of each well. The plates were incubated for 4 h at 37 °C, and the absorbance was recorded in a microplate reader (Biotek Elx800) at 570 nm for resofurin and 600 nm for resazurin. The percentage of cell viability was calculated by normalising the absorbance of each treatment with the NC, which was considered 100 %. All analyses were performed with four wells/replicates (*n* = 4) and three biological experiments (*n* = 3).

##### 2.6.3.3 Cell viability assay (3D spheroids)

The resazurin assay (Sigma-Aldrich) was performed according to Riss et al. (2011)and Walzl et al. (2014). After the initiation step, HepG2 and HT-29 spheroids were treated with DMEM (NC), DMSO (SC), or optimal BPC extract (100, 200 and 250 μg/mL) for 24, 48, 72, 120 and 168 h. Next, a resazurin working solution (0.15 mg/mL in PBS) was added to 20 % of the final volume of each well containing the tumour spheroids. The plates were incubated for 72 h at 37 °C, and the absorbance was recorded in a microplate reader (Biotek Elx800) at 570 nm for resofurin and 600 nm for resazurin. The absorbance values were multiplied by the oxidation factor at each wavelength and subtracted from converted resofurin. The percentage of cell viability was calculated by normalising the absorbance of each treatment with the NC, which was considered 100 %. All analyses were performed with six spheroids/replicates (*n* = 6) and three biological experiments (*n* = 3).

##### 2.6.3.4 Volume, morphology, and integrity analyses (3D spheroids)

The volume, morphology, and integrity of HepG2 and HT-29 spheroids were analysed based on the methods reported by Friedrich et al. (2009b) and Vinci et al. (2012). After the initiation step, photomicrographs of the HepG2 and HT-29 spheroids were recorded using the Axio Cam MRc image capture system (Carl Zeiss; Göttingen, Germany) coupled to an inverted Axio LabA1 microscope (Carl Zeiss) using the 4× objective and analysed with the aid of the AxioVision SE64 Rel. 4.9.1 software (Carl Zeiss). Then, the spheroids were treated with DMEM (NC), DMSO (SC), or optimal BPC extract (100, 200 and 250 μg/mL). For integrity/morphology analysis, the photomicrographs of each spheroid were obtained for 24, 48, 72, 120 and 168 h of treatment. In the integrity/morphology assessment, each image was analysed to detect irregular spheroids (without circular shape) or cell disaggregation. For cell volume quantification, the circumferences of tumour spheroids were analysed with the AxioVision SE Rel. 4.9.1 software using the “measure” tool. The area was reported in μm^3^ and the diameter in μm. All analyses were performed with six spheroids/replicates (*n* = 6) in three biological experiments (*n* = 3).

##### 2.6.3.5 Antiproliferative activity

The antiproliferative activity was assessed using the clonogenic assay described by Franken et al. (2006). Briefly, 1500 HepG2 cells were seeded in 35 mm diameter Petri dishes. Cultures were treated for 24 h with optimal BPC extract at 100, 200 and 250 μg/mL and recovered in fresh medium for 14 subsequent days. Then, the colonies were fixed and stained with Crystal Violet. Only colonies with >50 cells were counted using a manual haematological counter. Assays were performed in triplicate, and data were presented as mean ± SD of three independent experiments.

#### 2.6.4 Molecular docking

To verify the molecular interaction between the compounds in BPC extract and their potential protein target (Keap1, a repressor of Nrf2), molecular docking simulations were performed with AutoDock Vina (version 1.1.2). Keap1 is an inhibitory protein which binds to Nrf2 for degradation through the ubiquitin-proteasome pathway. The Keap1-Nrf2 complex prevents Nrf2 from activating gene expression related to the antioxidant response element. The 3D structure of Keap1 was obtained from the Protein Data Bank (PDB) with the entry of 4L7B, which includes the structure of the Keap1 kelch domain in a complex with a small molecule inhibitor. The PDB file was processed using PyMOL (version 2.5.5) to remove the built-in ligand, obtaining an isolated Keap1 structure. Polar hydrogens were added to the protein structure using AutoDock Tools (ADT) to generate the pdbqt file required for molecular docking. Additionally, the compounds from BPC were checked for rotatable bonds using ADT, and corresponding pdbqt files were subsequently generated. Based on the positional parameters of the original ligand in the PDB file, the optimal grid box parameters (center_x, center_y, center_z, size_x, size_y, and size_z) that fully encompassed the ligand's binding site were determined using the Grid box tool in ADT. In the Autodock Vina input file, the receptor and ligand structure files and the optimal grid box parameters were specified. Molecular docking simulations were conducted using Autodock Vina to calculate each BPC compound's optimal docking conformations and binding affinities with the Keap1 protein. The resulting 3D structures from the molecular docking simulations were visualised using PyMOL.

Each docking was repeated five times to avoid bias simulation. Using Eq. 4, the predicted inhibition constant (pKi) was calculated based on the mean of docking result (binding affinity):

(4) $$\mathrm{pKi}=-\log10\left[\exp\left(-\mathrm{\Delta G}/\mathrm{RT}\right)\right]$$

where ΔG is the binding affinity in kcal/mol, R is the gas constant (1.985e-3 cal/K/mol), and T is the temperature in Kelvin, which is set to be 298.15.

### 2.7 Application of blackcurrant press cake extract on functional gummies

#### 2.7.1 Gummy formulation

Ethics approval was granted by the University of Limerick (protocol: 2024_01_03_S&E) to create a functional gummy for sensory analysis. The manufacturing process occurred in a food-grade laboratory and involved preparing two separate gummy formulations: the BPC and control gummy. For both gummies, beef gelatine (10 g/100 g), bee honey (23 g/100 g), and flavouring agent (1 g/100 g) were used. For the BPC-infused gummy, inulin (5 g/100 g), xylitol (3 g/100 g), water (35 g/100 g), and dealcoholized BPC extract (23 g/100 g) were used in the formulation. In contrast, for the control gummy, sucrose (8 g/100 g), artificial colour agent bee honey (0.5 g/100 g), and water bee honey (57.5 g/100 g) were used in the formulation. In a 250 mL beaker, water, xylitol, honey, and inulin were combined and heated to 60 °C to homogenise the ingredients. Aluminium foil covered the beaker to inhibit the photo-oxidation of anthocyanins. Then, beef gelatine was added to the mixture and dissolved. The solution was then cooled to 40 °C before adding the flavouring agents to the control and BPC gummy mixture. At this point, BPC was also added to the BPC gummy solution. Before the mixture solidified, the mixture was poured into gummy moulds and frozen at −20 °C for 25 min. After freezing, the gummies were taken from the moulds and stored in a freezer for future use.

#### 2.7.2 Effects of in vitro static digestion on the chemical composition and antioxidant capacity of gummies

The in vitro INFOGEST digestion protocol followed the conditions proposed by Brodkorb et al. (2019),encompassing oral, gastric and intestinal digestions. Briefly, 5 g of gummies were weighed and mixed with 5 mL of water. The digestion was initiated by adding salivary fluid (pH 7) and α-amylase (14 U/mg) to 50 mL tubes and incubated for 2 min at 37 °C and 150 rpm. Then, gastric fluid (pH 3) and pepsin (25,000 U/mL) were added and incubated for 2 h (37 °C and 150 rpm). For the intestinal phase, intestinal fluid (pH 7) was added with pancreatin (800 U/mL) and bile salts (160 mmol/L), and the mixture was incubated for 2 h (37 °C and 150 rpm). After that, the enzymatic reaction was stopped by adding a 50 % trichloroacetic acid, and the tubes were centrifuged (12,000 x*g*, 10 min), and the supernatant was immediately analysed for chemical composition and antioxidant capacity. Blanks were also run parallel and analysed to account for any potential interferences from the digestion reagents. Eq. 5 was used to calculate the bioaccessibility, which represents the percentage of compounds released from blackcurrant press cake-added gummies considering the initial content (CI) and the content after the intestinal digestion (CA).

(5) $$\text{Bioaccessibility}\;\left(\%\right)=\mathrm{CA}/\mathrm{CI}\;x\;100$$

#### 2.7.3 Sensory analysis of gummies

The sensory analysis of gummies (control gummy produced using sugar and artificial colouring agent and the gummy added with optimal blackcurrant press cake extract) was conducted by 51 participants ranging from ages 18–48 in individual sensory booths with artificial white LED lights. Both gummies were presented monadically to the participants using a three-digit code and were evaluated using a 9-point hedonic scale (1 = disliked extremely, 5 = neither liked nor disliked, 9 = liked extremely) for attributes including colour, taste, texture, and overall impression. The acceptability index was calculated as a percentage, representing the degree of satisfaction relative to the highest possible score for “overall impression”. Eq. 6 was used to calculate gummies' acceptability index:

(6) $$\mathrm{Acceptability index}\;\left(\%\right)=\left(\frac{\mathrm{Average score}}{\mathrm{Maximum score}\;}\right)x\;100$$

Additionally, participants indicated their purchase intention of a gummy bag (100 g) on a 6-point scale (1 - I would not buy it; 2 – I would not pay anything more; 3 – I would pay €1.25; 4 – I would pay €1.50; 5 – I would pay €1.75; 6 – I would pay €2.00) considering the price of conventional gummy (€1).

### 2.8 Statistical analyses

Analyses were conducted in triplicate, with results presented as means ± standard deviation. Differences between groups were compared using a one-way analysis of variance (ANOVA), followed by Tukey's test for mean comparisons. For comparisons between two samples, a paired Student's *t*-test was applied to compare the initial results of in vitro digestion. Statistical analysis and graphical representation were performed using both GraphPad Prism software version 8.0.c (GraphPad Software, Inc., San Diego, CA, USA) and Microsoft Excel version 2011. A *p*-value of <0.05 was considered statistically significant.

## 3 Results and discussion

### 3.1 Extraction of bioactive compounds from blackcurrant press cake

#### 3.1.1 Effects of ultrasonic power and time on colour, antioxidant capacity, and chemical composition

According to Table 1, The TPC ranged from 11.32 to 20.21 mg GAE/g. The highest TPC was observed when the extraction was performed for 10 min at 400 W. In contrast, the lowest TPC was noted at 7.5 min/325 W. Additionally, at 400 W for 10 min, the highest TAC and antioxidant capacity (FRAP, DPPH, and plasma protection) were also achieved. It is evident from the assays that a higher ultrasonic power (e.g., 400 W) significantly (*p* < 0.05) enhanced the extraction of antioxidant compounds and the colour intensity from BPC. Holtung, Holtung et al. (2011) used UAE (45 kHz, 20 min; acetone; 1 g:4 mL) to extract BPC and found a TPC and TAC of 20 mg GAE/g and 6.86 mg CYE/g, respectively, whereas Pap et al. (2021a) used water and maceration (1 g: 10 mL, 60 min at 40 °C) and found a TPC and TAC of 9.44 mg GAE/g and 2.26 mg/g. Mohammadi, Franchin, et al. (2024) found a TPC and TAC of 4.63 mg GAE/g and 1.23 mg/g in blackberry fruit pulp extracted using UAE (400 W/10 min). Ultrasonic power increases cavitation, creating bubbles that enhance the mass exchange rate between BPC and the solvent.

**Table 1:** Chemical composition, instrumental colour, and antioxidant capacity of blackcurrant press cake extracts at various extraction conditions (time and ultrasonic power).

Ultrasonic power (W)	Extraction time (min)	TPC (mg GAE/g)	TAC (mg CYE/g)	FRAP (mg AAE/g)	DPPH (mg AAE/g)	Plasma protection (mg AAE/g)	Colour intensity (a.u.)	Yellow pigments (%)	Red pigments (%)	Blue pigments (%)
250	10	13.15 ± 0.70^b^	2.26 ± 0.05^ab^	23.70 ± 1.95^b^	10.36 ± 0.24^c^	16.35 ± 1.10^a^	3.07 ± 0.01^a^	32.24 ± 0.14^a^	55.87 ± 0.16^e^	11.89 ± 0.16^a^
400	5	11.52 ± 0.28^c^	2.03 ± 0.06^b^	19.61 ± 1.01^b^	10.45 ± 0.26^c^	15.47 ± 0.80^ab^	2.28 ± 0.02^d^	31.06 ± 0.09^b^	59.14 ± 0.11^d^	9.81 ± 0.03^b^
400	10	20.21 ± 0.50^a^	2.41 ± 0.21^a^	35.36 ± 1.02^a^	14.45 ± 0.53^a^	15.84 ± 0.77^a^	3.03 ± 0.04^a^	30.20 ± 0.07^c^	60.80 ± 0.10^c^	9.00 ± 0.03^d^
250	5	13.56 ± 0.41^b^	2.25 ± 0.11^ab^	22.86 ± 1.10^b^	13.64 ± 0.42^b^	13.26 ± 0.70^b^	2.54 ± 0.04^b^	27.76 ± 0.17^e^	62.84 ± 0.20^a^	9.40 ± 0.07^c^
325	7.5	11.32 ± 0.56^c^	2.25 ± 0.07^ab^	22.13 ± 2.72^b^	14.14 + 0.49^ab^	13.85 ± 1.81^ab^	2.40 ± 0.05^c^	28.48 ± 0.13^d^	62.02 ± 0.24^b^	9.51 ± 0.12^c^


Factors	Regression coefficients	Standard error	t-Value	p-Value	−95 % Confidence interval	+95 % Confidence interval
*Total phenolic content*						
Mean	13.90	0.12	117.20	<0.001	13.62	14.17
(1)Ultrasonic power	1.33	0.14	9.77	<0.001	1.01	1.64
(2)Time	2.03	0.14	15.01	<0.001	1.72	2.34
1 by 2	2.38	0.14	17.56	<0.001	2.07	2.70
R^2^	0.821					
R^2^ adj	0.762					
*Total anthocyanin content*						
Mean	2.24	0.03	65.31	<0.001	2.16	2.32
(2)Time	0.09	0.04	2.37	0.05	0.00	0.18
1 by 2	0.08	0.04	2.11	0.07	−0.01	0.17
R^2^	0.524					
R^2^ adj	0.428					
*FRAP*						
Mean	24.28	0.44	55.38	<0.001	23.27	25.29
(1) Ultrasonic power	2.24	0.50	4.45	<0.001	1.08	3.40
(2)Time	3.59	0.50	7.17	<0.001	2.43	4.74
1 by 2	3.86	0.50	7.68	<0.001	2.70	5.02
R^2^	0.885					
R^2^ adj	0.847					
*DPPH*						
Mean	12.64	0.10	130.24	<0.001	12.42	12.87
(1) Ultrasonic power	0.27	0.11	2.39	0.04	0.01	0.52
(2)Time	0.37	0.11	3.37	0.01	0.12	0.63
1 by 2	1.83	0.11	16.41	<0.001	1.57	2.08
R^2^	0.800					
R^2^ adj	0.733					
*Plasma protection*						
Mean	14.88	0.23	65.67	<0.001	14.36	15.40
(1) Ultrasonic power	0.50	0.26	1.94	0.09	−0.10	1.10
(2)Time	0.59	0.26	2.28	0.05	−0.01	1.18
1 by 2	−0.72	0.26	−2.79	0.02	−1.32	−0.12
R^2^	0.702					
R^2^ adj	0.603					
*Red pigments*						
Mean	60.20	0.05	1191.49	<0.001	60.09	60.32
(1) Ultrasonic power	0.32	0.06	5.47	<0.001	0.18	0.45
(2)Time	−1.24	0.06	−21.51	<0.001	−1.37	−1.11
1 by 2	2.17	0.06	37.51	<0.001	2.04	2.31
R^2^	0.820					
R^2^ adj	0.760					

Note: TPC = total phenolic content; TAC = total anthocyanin content; DPPH = 2,2-diphenyl-1-picrylhydrazyl; FRAP = Ferric ion reducing antioxidant power; GAE = gallic acid equivalent; AAE = ascorbic acid equivalent; CYE = cyanidin-3-glucoside equivalent.

Regarding plasma protection, all samples demonstrated the ability to protect human plasma against copper-induced plasma oxidation, with results ranging from 13.26 to 16.35 mg AAE/g. The lowest plasma protection was obtained using UAE at 250 W/5 min. Mohammadi, Franchin, et al. (2024) found plasma protection of 16.94 and 14.07 mg AAE/g in blackberry and elderberry fruit pulp extracts (UAE, 400 W/10 min), which are similar to the values reported for BPC). The plasma protection assay evaluates a substance's ability to shield plasma components, such as lipids and proteins, against oxidative damage caused by free radicals and/or other ROS. Investigating the antioxidant capacity and potential impacts of phenolic compounds on human blood is crucial for understanding their biological significance. This study aims to characterise BPC as a novel natural food ingredient, emphasising the importance of these compounds.

RSM models were proposed to evaluate the effects of extraction time and ultrasonic power on key parameters (Table 1). Both factors positively influenced the extraction of TPC, plasma protection, DPPH, red pigments, and FRAP, with their linear interaction being highly significant (*p* < 0.001). At higher ultrasonic power (400 W) and longer extraction times (10 min), the highest TPC, TAC, FRAP, and DPPH values were observed, suggesting a synergistic effect of power and time in enhancing extraction efficiency. A previous study on the extraction of phenolic compounds from purple tea showed that UAE outperformed maceration, yielding 3.5-fold higher TPC and antioxidant capacity, confirming its superior extraction efficiency (Mohammadi, Farrell, et al., 2024). Also, this observation highlights the importance of these extraction parameters for future scale-up processes. The RSM models accounted for 82.1 % of the variability in TPC, 70.2 % in plasma protection, 88.5 % in FRAP, 82.0 % in red pigments, and 80.0 % in DPPH, demonstrating their utility for predictive purposes. For TAC, extraction time and the interaction between time and ultrasonic power were the main factors (*p* < 0.001) affecting anthocyanin recovery. However, the RSM model for TAC explained only 52.4 % of data variability, rendering it unsuitable for prediction purposes. In conclusion, considering the data, the UAE conditions identified as optimal was 400 W/10 min.

#### 3.1.2 Characterisation of the optimal BPC extract

The optimal BPC extract's colour attributes were compared to a natural elderberry extract and a synthetic red pigment used in food applications. Table 2 shows that the optimal BPC extract has the second lowest redness (a* coordinate), whereas the synthetic food dye has the highest redness intensity. However, BPC extract has the highest (*p* < 0.05) percentage of blue pigments, probably because of anthocyanins. Colour intensity is paramount for sensory acceptability, and when it comes to producing new natural ingredients, comparison with synthetic colours is usually performed. Although the optimal BPC extract had lower redness than the synthetic red food dye, the colour intensity was not statistically different (4.16 vs 4.43, *p* > 0.05). Future studies should focus on the thermal and photostability of the optimal BPC extract.

**Table 2:** Colour attributes of blackcurrant press cake extract, elderberry natural colouring and synthetic red pigment.

Extract	L* (Lightness)	a* (redness)	b* (yellowness)	Yellow pigments (%)	Red pigments (%)	Blue pigments (%)	Colour intensity (a.u.)	RGB colour transformation	Real colour
*Blackcurrant press cake*	23.60 ± 0.94^c^	21.99 ± 1.88^c^	4.49 ± 0.80^b^	28.43 ± 1.18^c^	60.20 ± 4.59^b^	11.37 ± 3.40^a^	4.16 ± 0.80^a^		
*Elderberry natural colour*	49.50 ± 3.02^a^	40.03 ± 1.95^b^	−6.57 ± 0.95^c^	33.73 ± 0.06^a^	58.93 ± 0.27^b^	7.34 ± 0.24^b^	0.75 ± 0.01^b^		
*Synthetic red pigment*	45.90 ± 0.72^c^	56.99 ± 0.69^a^	48.39 ± 0.70^a^	30.86 ± 0.09^b^	69.16 ± 0.12^a^	−0.02 ± 0.03^c^	4.43 ± 0.21^a^		

The freeze-dried optimal BPC extract exhibited a TPC of 47.83 mg GAE/g, with anthocyanins constituting 35.69 mg CYE/g (75 % of TPC) (Table 3). The predominant anthocyanins identified were delphinidin-3-rutinoside (25.59 mg/g) followed by delphinidin-3-glucoside (5.31 mg/g), cyanidin-3-rutinoside (2.63 mg/g), and cyanidin-3-glucoside (2.16 mg/g). Additionally, the extract demonstrated a significant inhibitory effect on human plasma oxidation (5052 mg AAE/g), five times more effective than ascorbic acid. This enhanced effectiveness is likely due to its capacity to reduce and inhibit ROS redox-led reactions.

**Table 3:** Phenolic composition and antioxidant capacity of the freeze-dried optimal blackcurrant press cake extract obtained with ethanol:water (60:40 v/v) using ultrasound-assisted extraction at 400 W for 10 min. Typical chromatogram at 520 nm (DAD) of the optimal blackcurrant press cake extract obtained by ultrasound-assisted extraction. Peaks identification 1- Delphinidin-3-glucoside, 2- Delphinidin-3-rutinoside, 3- Cyanidin-3-glucoside, 4- Cyanidin-3-rutinoside.

Composition and bioactivity	Freeze-dried extract
*Phenolic composition*	
Total phenolic content (mg GAE/g)	47.83 ± 1.04
Delphinidin-3-rutinoside (mg/g)	25.59 ± 0.01
Cyanidin-3-rutinoside (mg/g)	2.63 ± 0.01
Cyanidin-3-glucoside (mg/g)	2.16 ± 0.01
Delphinidin-3-glucoside (mg/g)	5.31 ± 0.05

*Antioxidant capacity*	
Iron-reducing capacity (mg GAE/g)	14.40 ± 1.27
Ferric-reducing antioxidant power (mg AAE/g)	71.37 ± 0.55
DPPH free radical scavenging activity (mg AAE/g)	98.79 ± 11.31
Inhibition of human plasma oxidation (mg AAE/g)	5052 ± 586

Note: GAE = gallic acid equivalent, AAE = ascorbic acid equivalent.

#### 3.1.3 Anthocyanins' reversibility toward pH shifts

**Fig. 1:**
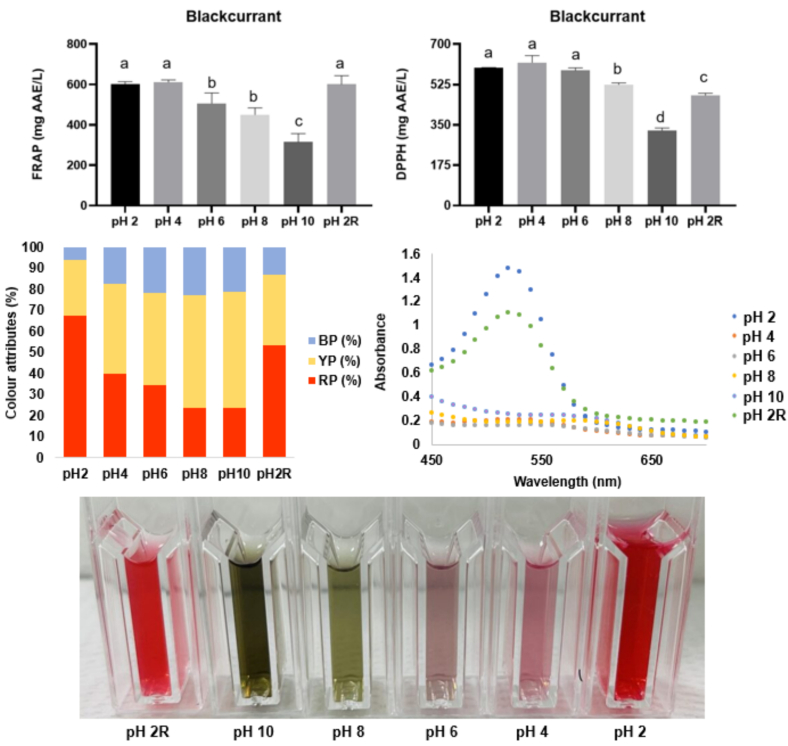
Effect of various pH values (2, 4, 6, 8, and 10) on the colour attributes and antioxidant capacity of blackcurrant press cake extract obtained with ethanol:water (60:40 v/v) using ultrasound-assisted extraction at 400 W for 10 min.

Regarding the effects of various pH values (pH 2 to pH 10) on the chemical stability, antioxidant capacity, and colour attributes of the liquid optimal BPC extract (Fig. 1), it is possible to observe that the redness peaks at pH 2. From pH 4 to 10, the percentage of red pigments decrease with an increased yellow pigment, typical of (partial) degradation. Additionally, the lowest redness and antioxidant capacity measured by the DPPH and FRAP assays peaks at pH 10. The colour shifts from intense red-pinkish (pH 2) to dark green (pH 10), with significant (*p* < 0.05) changes from pH 4 (FRAP) or pH 6 (DPPH) to pH 10. When the reverse process of protonation/deprotonation was performed (pH 10 to pH 2R), anthocyanins exhibited 79.3 % reversibility using the percentage of red pigments (e.g., absorbance at λ = 520 nm). Concerning the effects of pH shifts on the antioxidant capacity (FRAP), the highest averages were observed at pH 2 and 4, and the deprotonation/protonation process did not impact the reversibility (e.g., reversibility rate of 99.6 %), as FRAP values at pH 2 and pH 2R are similar (*p* > 0.05). For DPPH, the highest averages were observed for pH 2, pH 4, and pH 6, and the protonation/deprotonation process significantly decreased the antioxidant capacity, as pH 2 and pH 2R are different (p < 0.05) with a reversibility rate of 80.1 %.

In low pH values (*p* < 4.5), flavylium cation stands out, and anthocyanins are highly stable. However, when NaOH is added to the solution, anthocyanins can be partially decomposed by proton transfer, hydration and tautomerisation reactions, forming quinoidal bases (Sousa et al., 2022). For food processing, most foods and beverages are acidic. Thus, the degradation of anthocyanins is not expected to occur as fast as in the model system tested herein. Black rice extract, rich in cyanidin-3-glucoside, exhibited 50 % reversibility, lower than the value reported for the optimal BPC extract (Pedro et al., 2016). In contrast, elderberry and blackberry extracts, rich in cyanidin-3-glucoside, also showed a reversibility rate of 100 and 98 %, respectively, higher than the value reported for the optimal BPC extract.

### 3.2 Effects of the optimal BPC extract on intracellular ROS generation in human erythrocytes

**Fig. 2:**
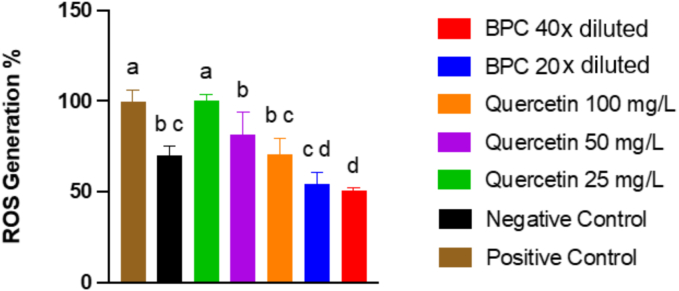
Effect of blackcurrant press cake extract on intracellular reactive oxygen species (ROS) generation induced by AAPH (2,2′-azobis(amidinopropane) dihydrochloride). Different letters represent statistical differences (p < 0.05). *BPC; optimal blackcurrant press cake extract.

The optimal dealcoholised BPC extract (diluted 20- and 40-fold) effectively reduced intracellular ROS generation induced by peroxyl (ROO^•^) and alkoxyl (RO^•^) radicals formed by the thermal decomposition of AAPH by 54 % and 51 %, respectively, compared to the positive control (Fig. 2). This antioxidant activity is possibly associated with the extract's phenolic compounds, especially anthocyanins (Zhang et al., 2023). The antioxidant activity of blackcurrant extracts has been previously demonstrated in lipopolysaccharide-stimulated RAW264.7 macrophages, where the blackcurrant extract (100 μg/mL) decreased intracellular ROS generation to 30 % (Hui et al., 2021). In our previous study, BPC was digested using an in vitro protocol (e.g., INFOGEST), and the undigested and the digested BPC extracts were able to reduce intracellular ROS generation in human erythrocytes in a dose-dependent manner (50 to 100 μg GAE/mL) (dos Santos Lima et al., 2024b). These findings highlight the potential of BPC extracts to mitigate oxidative stress in human erythrocytes, emphasising the importance of phenolic compounds, especially anthocyanins, in their protective mechanisms.

### 3.3 Modulation of the cellular antioxidant and anti-inflammatory response in THP-1 macrophage cultures: Molecular docking simulation and in vitro validation

**Fig. 3:**
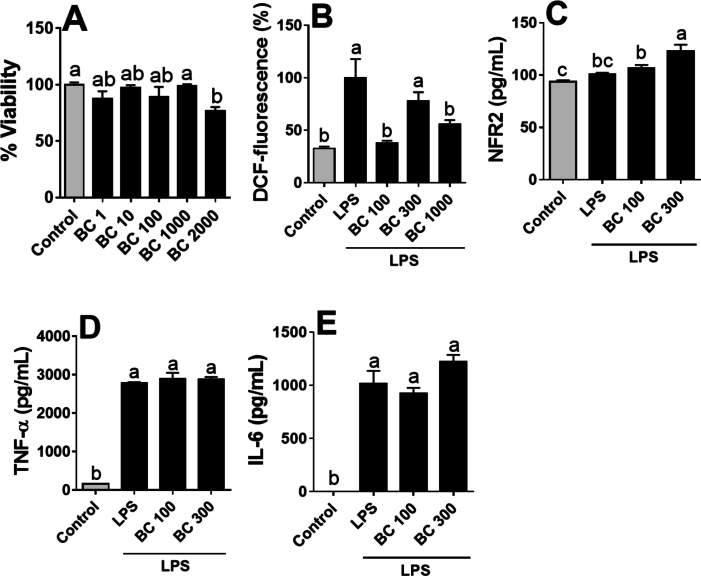

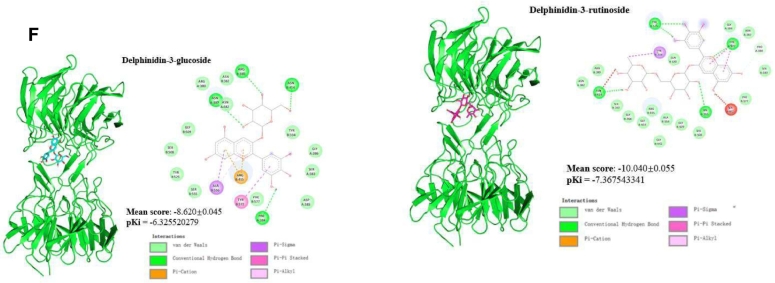

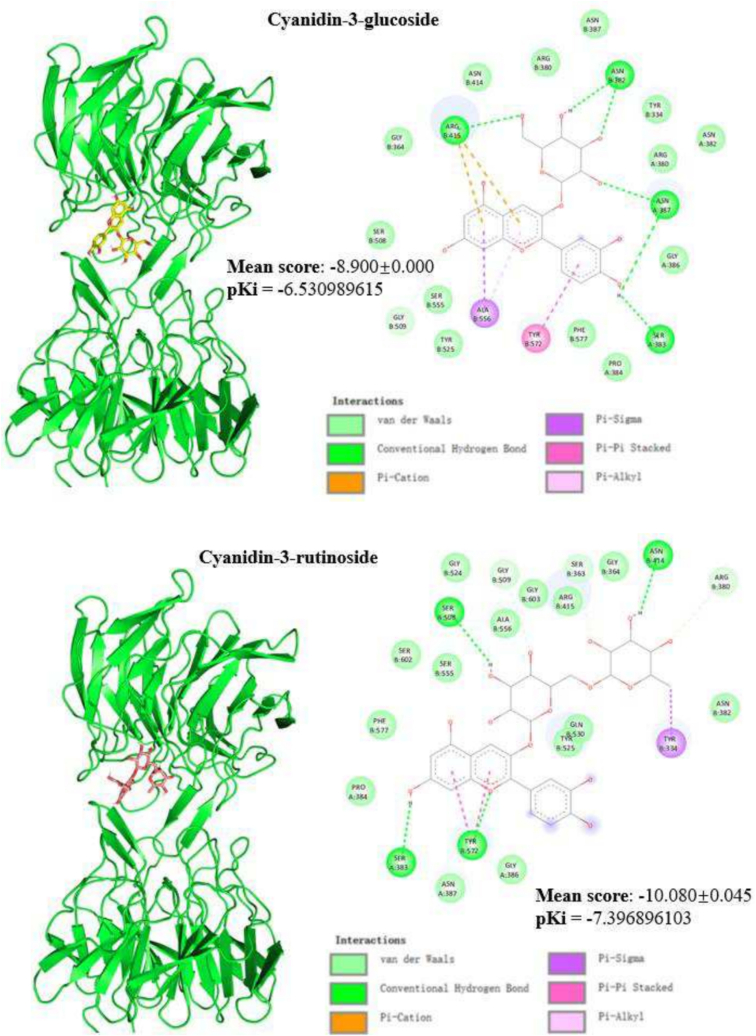
Effect of the optimal BPC extract on the antioxidant and anti-inflammatory response in THP-1 macrophages. (A) Cell viability using the MTT assay; (B) Quantification of intracellular reactive oxygen species (ROS) caused by H_2_O_2_ treatment and the antioxidant effects of BPC extract; (C) Nuclear factor erythroid 2-related factor 2 (Nrf2) quantification; (D) Tumour necrosis factor-alpha (TNF-α) quantification; (E) Interleukin-6 (IL-6) quantification; (F) 3D and 2D binding poses of BPC anthocyanins and Keap1. Different lowercase letters denote significant differences between treatments at 95 % confidence. Quantitative data are the mean ± standard deviation (n = 3–5).

Initial viability studies of THP-1 macrophage-like cells exposed to increasing concentrations of optimal BPC extract (Fig. 3A) indicated significant toxicity at a dose of 2000 μg/mL (23.1 % viability reduction) compared to the control group (*p* < 0.05). No cytotoxicity was observed between 1 and 1000 μg/mL (*p* > 0.05). Based on these findings, 100, 300, and 1000 μg/mL concentrations were selected for subsequent experiments in THP-1 cell cultures.

The generation of free radicals in THP-1 macrophage-like cells was evaluated using the classic DCFH-DA assay. As shown in Fig. 3B, LPS stimulation led to a 67.5 % increase in ROS production compared to the negative control group (p < 0.05). The antioxidative potential of BPC was confirmed upon co-treatment with LPS-stimulated THP-1 macrophage-like cells. The optimal BPC extract at 100 and 1000 μg/mL concentrations significantly reduced ROS production by 62 % and 45 %, respectively, after 6 h (p < 0.05). The antioxidant capacity of the BPC extract was further validated by increased Nrf2 protein expression levels in LPS-stimulated THP-1 macrophage-like cells compared to the LPS-only group (p < 0.05) (Fig. 3C). Nrf2 is a transcription factor that serves as a sensor for oxidative stress and stimulates antioxidant cellular responses. Various studies have highlighted BPC's capacity as a potent antioxidant (Gopalan et al., 2012). Recent investigations by dos Santos Lima et al. (2024b) and Pap et al. (2021b) have confirmed crude BPC extract's ability to suppress intracellular ROS generation following H_2_O_2_ challenge in multiple in vitro cellular model systems, including Caco-2, HepG2, EA.hy926, and HCT8 cells. Furthermore, in the dos Santos Lima et al. (2024c) study, DPC treatment reduced lipid peroxidation levels in EA.hy926 cells, indicating a potential protective role against oxidative membrane damage.

Finally, the production of inflammatory cytokines was evaluated in THP-1 macrophage-like cells that were stimulated with 100 ng/mL LPS and pre-treated with the optimal BPC extract at 100 and 300 μg/mL. The results indicated that LPS stimulation for 6 h or 24 h significantly increased the secretion of TNF-α (17-fold) and IL-6 (10-fold), respectively (Fig. 3D and E, *p* < 0.05). However, pre-treatment with the BPC extract did not significantly modulate the secretion of either cytokine (*p* > 0.05), indicating a lack of anti-inflammatory effects under these conditions. Previous studies have shown that BPC can reduce mRNA levels of pro-inflammatory cytokines (IL-6 and IL-1β) in RAW 264.7 macrophages and suppress the expression of M1-like pro-inflammatory markers in murine bone marrow-derived macrophages (Lee et al., 2019). The lack of anti-inflammatory effects of the BPC extract may be attributed to differences in cellular responses, as THP-1 macrophages may react differently compared to RAW 264.7 cells or murine macrophages used in previous studies. Additionally, the strong inflammatory response induced by LPS (a 17-fold increase in TNF-α and a 10-fold increase in IL-6) could have overwhelmed the extract's potential effects. Further studies are needed to clarify these initial results.The molecular docking outputs shown in Fig. 3F revealed probable binding poses of the compounds in BPC extract and Keap1. The binding affinities of the four compounds ranged from −8.6 to −10.1 kcal/mol, indicating that the molecular interactions between BPC anthocyanins and Keap1 are strongly significant. In Fig. 3F, the 3D binding poses demonstrate that all the compounds are in the Kelch domain, and the 2D figures show the specific residues interacting with the ligands. Hydrogen bonds significantly increase the stability of the molecular interactions. Ligands with such a high binding affinity competitively inhibit Keap1, contributing to the activation of Nrf2 and other transcription of antioxidant response element-driven genes. In the context of redox homeostasis, Keap1, a cytosolic repressor, plays a pivotal role in regulating the activity of Nrf2. Under normal conditions, the Kelch domain of Keap1 will bind to Nrf2, facilitating the ubiquitination and subsequent proteasomal degradation of Nrf2. Therefore, the activity of Nrf2 is restricted. However, the anthocyanins from BPC extract (delphinidin-3-rutinoside, delphinidin-3-glucoside, cyanidin-3-rutinoside, and cyanidin-3-glucoside) are predicted to bind to the Kelch domain of Keap1, competitively inhibiting the Keap1-Nrf2 interaction (Li et al., 2019).

As demonstrated in Fig. 3C, BPC extract significantly enhanced Nfr2 activation in a dose-dependent manner from 100 to 300 μg/mL (*p* < 0.05), corroborating the accuracy of the molecular docking simulation. The activation of Nrf2 is linked to the upregulation of cellular defence mechanisms against oxidative stress, inflammation, and other stressors. Therefore, these findings suggest that the consumption of BPC extract could confer substantial health benefits by promoting Nrf2 activation and bolstering the body's defence against oxidative stress-related diseases (Tu et al., 2019).

### 3.4 Cytotoxic and antiproliferative activity of blackcurrant press cake extract in monoculture and 3D spheroid

**Fig. 4:**
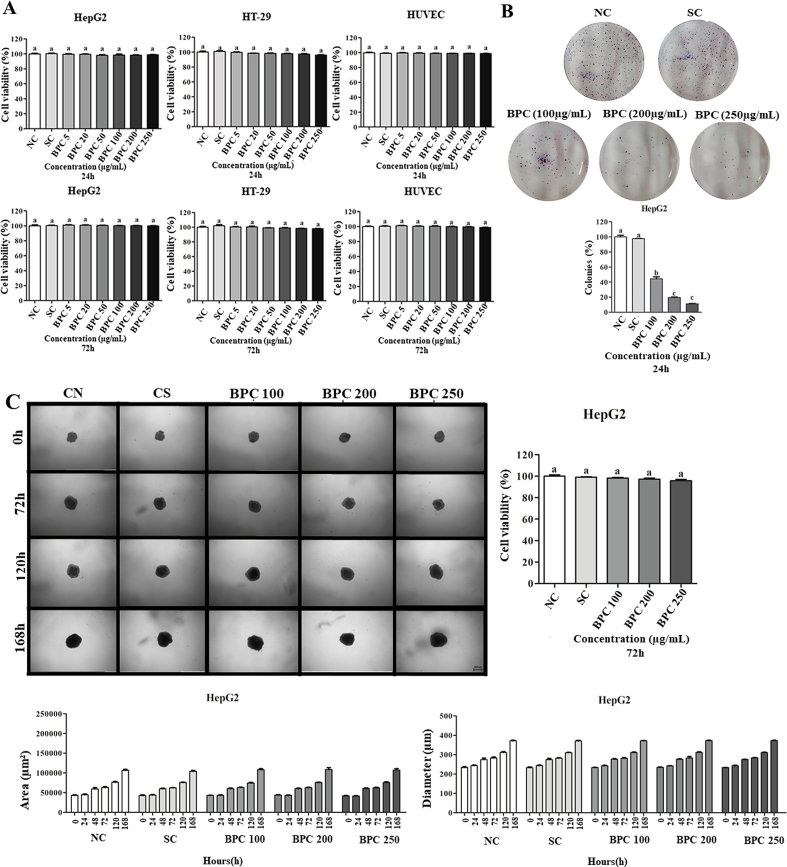

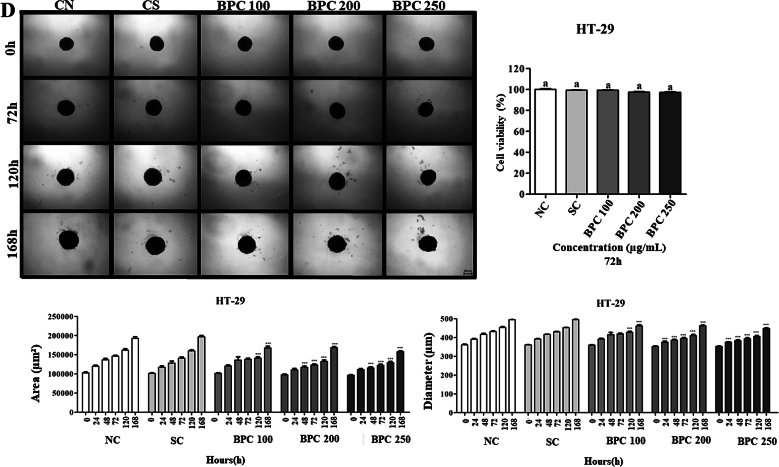
(A) Cell viability was determined by resazurin assay after 24 h and 72 h of treatment with optimal BPC extract (5–250 μg/mL). DMSO (0.08 % *v*/v) was used as solvent control. (B) Clonogenic assay of HepG2 cells treated with BPC 100, 200 or 250 μg/mL for 24 h and recovered in fresh medium for 14 days. (C—D) Morphology, integrity, área (μm^2^) and diameter (μm) of HepG2 and HT-29 spheroids after treatment with BPC for 0 (Day 4), 72 (Day 7), 120 (Day 9), and 168 h (Day 11). All images were obtained using the Axio Cam MRc capture system coupled to Axio LabA1 microscope, using the 4× objectives. Means with the same letter do not vary from each other (p > 0.05), and different letters comparing treatments represent statistically different results (*p* < 0.05). NC: negative control (culture medium); SC: DMSO 0.08 %. Scale: 200 μm.

In the present study, freeze-dried optimal BPC extract's cytotoxic and cell viability activities were evaluated at various concentrations (5–250 μg/mL). For the monoculture assays, hepatocellular carcinoma (HepG2) and colorectal adenocarcinoma (HT-29) tumour cell lines were used. Human umbilical vein endothelial cell (HUVEC) as a non-tumour cell model to evaluate the selectivity of the extract. According to the results presented in Fig. 4A, the viability of HUVEC, HepG2 and HT-29 cells treated with BPC at 5–250 μg/mL remained unchanged (IC_50_ > 250 μg/mL). The present results show that the extract did not exert a cytotoxic effect on the cell lines at any treatment time (24 and 72 h). Based on the American National Cancer Institute (NCI) and Geran's protocol, cytotoxicity can be classified as follows: IC_50_ ≤ 20 μg/mL indicates a highly cytotoxic extract, an IC_50_ value between 21 and 200 μg/mL indicates a moderately cytotoxic extract, and an IC_50_ value between 201 and 500 μg/mL indicates a weakly cytotoxic extract. Conversely, an IC_50_ value greater than 501 μg/mL indicates a non-cytotoxic extract (Goldin et al., 1981; Grever et al., 1992). dos Santos Lima et al. (2024d) tested crude blackcurrant extract and found no cytotoxic or cell viability activity in all cell lines tested (Caco-2, EA.hy926 and HepG2), as in our study. Another cell proliferation study, performed by Wu et al. (2007),in HT-29 cells treated with 0–60 mg/mL BPC extract showed significant inhibition of cell growth at 20 mg/mL (20 % reduction in cells).

Given that the optimal BPC extract did not exhibit cytotoxic effects in monoculture-treated cells, we extended our evaluation to assess its cytotoxic activity and impact on cell viability in HepG2 and HT-29 cell spheroids (3D cultures). The cells were treated with BC at 100, 200, and 250 μg/mL concentrations over 72 h. We also examined the potential volume, morphology, and structural integrity alterations of the HepG2 (Fig. 4C) and HT-29 (Fig. 4D) spheroids. The findings revealed that the viability of both HepG2 and HT-29 spheroids treated with BPC remained comparable to that observed in monoculture conditions, indicating no cytotoxic effects. Specifically, for HepG2 spheroids (Fig. 4C), there were no significant changes in area and diameter measurements relative to the control spheroids. In contrast, HT-29 spheroids (Fig. 4D) demonstrated a reduction in both area and diameter at BPC concentrations of 200 and 250 μg/mL, particularly notable after 24 h (diameter) and 48 h (area) of treatment. Additionally, at 120 and 168 h, the HT-29 spheroids exhibited signs of disaggregation and structural disintegration. Furthermore, it is noted that the HT-29 spheroids, at times 120 and 168 h, begin to disaggregate and fall apart. To our knowledge, this is the first study to investigate the effects of blackcurrant treatment on cell spheroids in a 3D culture model. No prior studies have reported similar findings, underscoring the novel nature of this research.

Given that the optimal BPC extract did not affect the viability or cytotoxicity of HepG2 and HT-29 cells, either in monoculture or spheroids, the antiproliferative activity of the sample was evaluated on the HepG2 cell line, using the clonogenic assay (Fig. 4B). The HepG2 cell line was selected due to its expression of phase I and II enzymes involved in drug metabolism processes, making it a metabolically active line (Valentin-Severin et al., 2003). The clonogenic assay results indicated a significant reduction in colony formation frequency, with reductions to 44 % (100 μg/mL), 19 % (200 μg/mL) and 11 % (250 μg/mL) in cells treated with BPC for 24 h, compared to the control groups (Fig. 4B). The results demonstrate that the BPC extract has antiproliferative activity and cytostatic effect on HepG2 cells in monoculture. Regarding HT-29 spheroids, BPC extract significantly impacted the area and diameter measurements of the 3D cultures, indicating an inhibitory effect on spheroid growth. These results suggest that the optimal BPC extract possesses potent antiproliferative properties, warranting additional investigation into its potential therapeutic applications.

### 3.5 Sensory evaluation of functional gummies

**Fig. 5:**
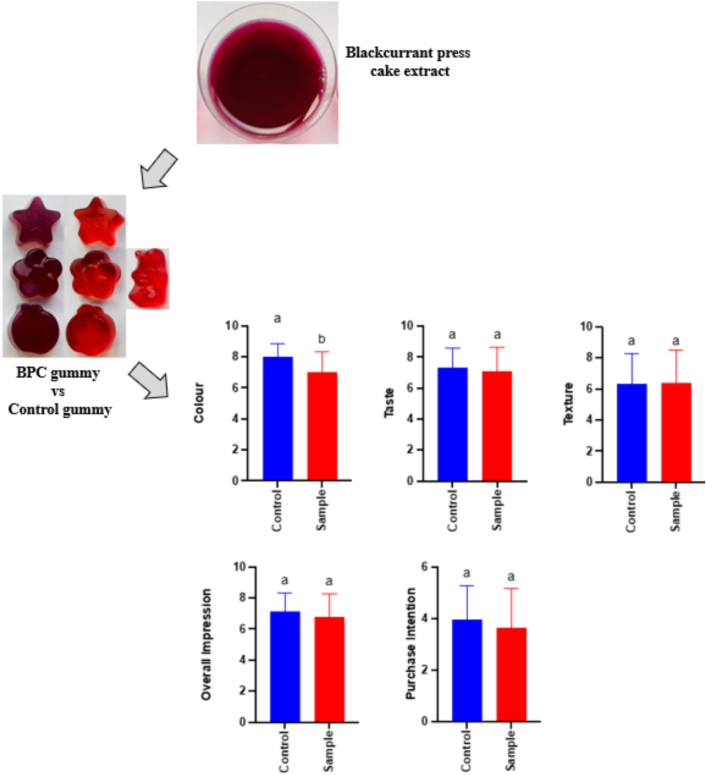
Sensory evaluation of colour, texture, taste, overall impression and purchase intention in BPC gummies (**red** bars) and control gummies (**blue** bars). Colour, texture, taste and overall impression correspond to a 9-point hedonic scale, while purchase intention corresponds to a 6-point hedonic scale. Different lower-case letters indicate significant (p < 0.05 differences between the media of each sample. (For interpretation of the references to colour in this figure legend, the reader is referred to the web version of this article.)

The 9-point hedonic scale was utilized to assess consumer acceptance of two gummy formulations: a control gummy with artificial colouring and a naturally sweetened BPC gummy. As shown in Fig. 5, except for colour, no statistically significant differences (*p* > 0.05) were observed in the mean scores between the control and BPC gummies, meaning that the BPC extract did not negatively impact on the gummy's sensory traits. The control gummy consistently achieved a higher average score for colour, likely due to consumer preference for vibrant red hues, which are commonly associated with familiarity and sweetness. The BPC and the control gummy garnered an acceptability rate of 76 % and 79 %, respectively, which is non-significant (p > 0.05). In terms of taste, although not significant (p > 0.05), the control gummy received a slightly higher average score (7.3) compared to the BPC gummy (7.1), likely due to the use of artificial sweeteners in the former, which are often preferred over natural alternatives. Texture scores were similar but suboptimal for both samples (below the acceptable threshold of 7), indicating the need for further optimisation.

Regarding the purchase intention, BPC and the control gummy garnered an average score of 3.6 and 3.9 out of 6, respectively (Fig. 5) and did not differ significantly (p > 0.05). In fact, 37 out of 50 panellists (e.g., 74 %) would pay at least 25 % more (e.g., ≥€1.25) for the BPC-infused gummy, and 44 out of 50 panellists (e.g., 88 %) would pay at least 25 % more (e.g., ≥€1.25) for the control gummy. Although the control gummy had higher average scores for most sensory attributes, the results for the BPC-infused gummies are promising and underscore the viability of the BPC gummy as an appealing and potentially functional alternative to conventional gummy formulations made of synthetic food dyes and sucrose.

### 3.6 Effects of in vitro digestion on the bioaccessibility of antioxidants from gummies

**Fig. 6:**
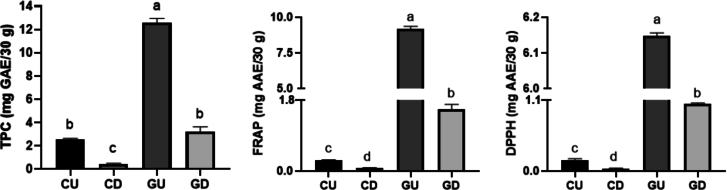
Effect of in vitro gastrointestinal digestion on 30 g samples of control gummies and BPC gummies. TPC (mg GAE/ 30 g), DPPH (mg AAE/30 g), and FRAP (mg AAE/30 g) were analysed pre- and post-digestion. Results are the means of four determinations ± standard deviation. Values with different lower-case letters are statistically different, as determined by a paired Student-*t-*test (p < 0.05). Note: TPC = total phenolic content; DPPH = 2,2-diphenyl-1-picrylhydrazyl; FRAP = Ferric ion reducing antioxidant power. CU = control - undigested; CD = control - digested, GU = BPC-infused gummy - undigested; GD = BPC-infused gummy - digested.

Fig. 6 reveals significant differences in the total phenolic content (TPC) and antioxidant capacity between the BPC-infused gummy and the control gummy after in vitro digestion. The BPC-infused gummy exhibited significantly higher (*p* < 0.05) TPC, FRAP, and DPPH values compared to the control gummy, demonstrating that the blackcurrant extract effectively enhanced the gummy's antioxidant capacity.Both gummies showed a significant reduction in TPC post-digestion, but the BPC gummy retained slightly higher levels of bioaccessible phenolics (26 %) compared to the control gummy (17 %), suggesting that natural anthocyanin-rich ingredients in the BPC gummy may have a positive impact on phenolic retention through digestion. Anthocyanins are associated with several health benefits due to their cellular antioxidant activity. In particular, cyanidin and delphinidin, the primary anthocyanidins in blackcurrants, are known to exhibit potent free radical-scavenging properties in vivo (Mohammadi, Farrell, et al., 2024). The antioxidant capacity measured by the FRAP and DPPH radical-scavenging assays further illustrated the antioxidant potential of the BPC-infused gummy. Both assays indicated higher initial antioxidant activity in the BPC gummy compared to the control. In fact, the antioxidant capacity of the control gummy is negligible (<1 mg AAE/30 g). Although digestion reduced the antioxidant capacity of both gummies, the BPC gummy's antioxidant potential remained superior post-digestion compared to the control gummy. For instance, BPC-infused gummy had only 17 % bioacessibility for both FRAP and DPPH. Regarding anthocyanins' bioacessibility, experimental results showed values lower than <0.5 mg/30 g (data not shown). In future experiments, concentrating the BPC extract, possibly by freeze-drying, could expand its application in gummies by increasing the anthocyanin content.

These findings support the BPC gummy's potential as a functional food, positioning it as a valuable product for delivering bioactive compounds. The results underscore the BPC gummy's role in meeting consumer demand for natural, health-promoting ingredients with scientifically demonstrated functional properties.

### 3.7 Limitations

While in vitro assays are invaluable for initial screening and mechanistic insights, the results should be interpreted cautiously when predicting functional food benefits. Future studies should focus on validating our findings (e.g., antioxidant capacity, anti-inflammatory activity, erythrocyte and plasma protection) in vivo, considering the complexities of bioavailability, food matrices, and systemic distribution and interactions to provide a more robust foundation for the development of BPC-infused functional foods.

## 4 Conclusions

An optimal food-grade blackcurrant press cake extract rich in anthocyanins was obtained using ultrasound-assisted extraction at 400 W for 10 min. Anthocyanins accounted for 75 % of total phenolics in the extract, with delphinidin-3-rutinoside being the predominant compound. The anthocyanins demonstrated partial reversibility (approximately 80 %) when the pH was altered from 2 to 10 and then back to pH 2, indicating relative chemical stability suitable for incorporation into acidic foods and beverages. The optimal extract exhibited antioxidant capacity in both chemical and biological media, in different human-derived cell lines, such as erythrocytes and THP-1 macrophage-like cells. However, no pro-inflammatory cytokines (IL-6, TNF-α) downregulation was observed in LPS-challenged THP-1 macrophage-like cells. Additionally, the optimal blackcurrant extract demonstrated antiproliferative activity and a cytostatic effect on HepG2 cells in monoculture, without compromising the viability of normal cells, such as endothelial HUVECs. When incorporated into a functional food product, such as gummies, the optimal extract increased the total phenolic content without adversely affecting taste and sensory acceptability. In vitro digestion studies revealed that the bioaccessibility of total phenolic contant and antioxidant capacity (e.g., FRAP and DPPH) decreased significantly. In conclusion, the food-grade extract developed in this study shows potential for functionalising gummy formulations. Further in vitro and in vivo testing of these formulations is recommended to maximise the use and reuse of blackcurrant press cake.

## CRediT authorship contribution statement

**Emma Brennan:** Writing – review & editing, Formal analysis, Data curation. **Carolina Girotto Pressete:** Writing – original draft, Formal analysis, Data curation. **Nima Mohammadi:** Writing – original draft, Formal analysis, Data curation. **Lusânia Maria Greggi Antunes:** Writing – review & editing, Resources, Project administration, Methodology. **Qixiang Shang:** Writing – original draft, Investigation, Formal analysis. **Jihang Chen:** Writing – review & editing, Methodology. **Jason Bennett:** Writing – review & editing, Methodology, Funding acquisition, Formal analysis. **Marcelo Franchin:** Writing – original draft, Methodology, Investigation, Formal analysis. **Daniel Granato:** Writing – original draft, Supervision, Software, Resources, Project administration, Methodology, Funding acquisition, Formal analysis, Data curation, Conceptualization.

## Declaration of competing interest

The authors declare that they have no known competing financial interests or personal relationships that could have appeared to influence the work reported in this paper.

## Data Availability

Data will be made available on request.
